# Toll-like Receptor 4 Is Upregulated in Parkinson’s Disease Patients and Co-Localizes with pSer129αSyn: A Possible Link with the Pathology

**DOI:** 10.3390/cells12101368

**Published:** 2023-05-11

**Authors:** Carmela Conte, Angela Ingrassia, John Breve, John J. Bol, Evelien Timmermans-Huisman, Anne-Marie van Dam, Tommaso Beccari, Wilma D. J. van de Berg

**Affiliations:** 1 Department of Pharmaceutical Sciences, University of Perugia, 06100 Perugia, Italy; tommaso.beccari@unipg.it; 2 Department of Anatomy and Neurosciences, Amsterdam Neuroscience, Amsterdam UMC, Vrije Universiteit Amsterdam, 1081 HZ Amsterdam, The Netherlands; a.ingrassia@amsterdamumc.nl (A.I.); jjp.breve@amsterdamumc.nl (J.B.); jgjm.bol@amsterdamumc.nl (J.J.B.); e.timmermans@amsterdamumc.nl (E.T.-H.); amw.vandam@amsterdamumc.nl (A.-M.v.D.)

**Keywords:** Parkinson’s disease, neuroinflammation, alpha-synuclein, substantia nigra pars compacta, middle temporal gyrus

## Abstract

Growing evidence suggests a crucial role of neuroinflammation in the pathophysiology of Parkinson’s disease (PD). Neuroinflammation is linked to the accumulation and aggregation of a-synuclein (αSyn), the primary pathological hallmark of PD. Toll-like receptors 4 (TLR4) can have implications in the development and progression of the pathology. In this study, we analyzed the expression of TLR4 in the substantia nigra (SN) and medial temporal gyrus (GTM) of well-characterized PD patients and age-matched controls. We also assessed the co-localization of TLR4 with pSer129 αSyn. Using qPCR, we observed an upregulation of TLR4 expression in the SN and GTM in PD patients compared to controls, which was accompanied by a reduction in αSyn expression likely due to the depletion of dopaminergic (DA) cells. Additionally, using immunofluorescence and confocal microscopy, we observed TLR4-positive staining and co-localization with pSer129-αSyn in Lewy bodies of DA neurons in the SN, as well as in pyramidal neurons in the GTM of PD donors. Furthermore, we observed a co-localization of TLR4 and Iba-1 in glial cells of both SN and GTM. Our findings provide evidence for the increased expression of TLR4 in the PD brain and suggest that the interaction between TLR4 and pSer129-αSyn could play a role in mediating the neuroinflammatory response in PD.

## 1. Introduction

Parkinson’s disease (PD) is the second most common neurodegenerative disorder. It is characterized by massive, slow and progressive loss of dopaminergic and other monoaminergic neurons, as well as the presence of alpha-synuclein (αSyn) immunoreactive Lewy bodies (LBs) and Lewy neurites (LNs) in the brain. The cardinal signs of PD are motor symptoms such as resting tremor, muscular rigidity and bradykinesia. In addition to motor manifestations, non-motor symptoms, such as mood disorders, insomnia, constipation, olfactory dysfunctions, dizziness and fainting are often present, even in the prodromal phase of PD [1,2]. The International Parkinson Disease and Movement Disorders Society has proposed new standardized diagnostic criteria for prodromal PD, leading to a new definition that includes a clinicogenetic subtype of PD and recognizes multiple stages of early disease [3]. The exact factors underlying PD pathogenesis and disease progression are unknown, but increasing evidence suggests a crucial role for chronic inflammation and immune response [4,5]. Neuroinflammation is a complex process involving reactive and extensive gliosis in different brain regions, including the substantia nigra pars compacta (SN*pc*), that leads to the release of pro-inflammatory molecules by microglial cells and to the activation and infiltration of circulating leukocytes in the brain, which may contribute to PD pathology [6,7,8,9,10,11].

It is well known that the activation of the toll-like receptor (TLR) signaling pathway plays a key role in neuroinflammation and subsequent neurodegeneration [12,13]. TLRs are a group of transmembrane receptors widely distributed in the CNS and they are involved in neurodegenerative diseases such as Alzheimer disease, PD and amyotrophic lateral sclerosis. The upregulation of TLRs during PD can lead to an overactivation of innate inflammatory processes and exacerbation of neuronal cell death [14,15]. TLR signaling is triggered by sensing/recognition of pathogen-associated molecular patterns (PAMPs) and/or by endogenous damage-associated molecular patterns (DAMPs) resulting in priming and activation [16,17]. In some cases, the activation of microglial TLR signaling can be beneficial, such as when it helps to clear and degrade pathological proteins such as αSyn or amyloid in AD [18,19,20]. Therefore, both helpful and harmful effects can result from TLR activation.

Reports suggest that αSyn can directly act as a DAMP for TLRs and lead to changes in TLRs expression, microglial activation, and the release of inflammatory mediators that may harm adjacent neurons [21,22]. However, due to the complexity of the immune system and the numerous factors that affect neuroinflammation, it is premature to define the exact role of TLRs. Nevertheless, changes in TLRs signaling have been observed in the SN*pc* of PD patients compared to controls and are believed to contribute to microglia-induced neuroinflammation [13]. Among TLR members, TLR2 [12,23,24,25,26], TLR4 [13,24,27,28,29,30,31] and TLR9 [32,33] have been specifically linked to the pathogenesis of PD. To understand the role of the TLR family in PD, more research is needed on TLRs in both healthy and diseased aging brains. Since discordant studies describe either beneficial or detrimental effects of TLRs in the CNS, additional studies addressing this question are warranted.

The current study aims to investigate the expression of TLR4 in PD. Studies indicate that TLR4 can interact with PD-associated αSyn [29]. Furthermore, there is evidence supporting the role of TLR4 in αSyn triggered pathology. TLR4 ablation has been shown to impair microglial phagocytosis and the clearance of αSyn, resulting in an acceleration of dopaminergic neuronal death [20,27,34]. We conducted a detailed analysis of TLR4 expression in SN and GTM brain regions using qPCR. TLR4 expression is believed to be altered by αSyn [21], and therefore we also measured αSyn mRNA levels. Although its role is debated, pS129 modification occurs during α-syn aggregation and participates in the initiation and progression of disease [35]. In addition, studies reported Iba1^+^ microglia in the postmortem PD nigra [23]. Here, we assessed the association between TLR4 and pSer12-αSyn and between TLR4 and Iba1 in the PD(D) brain using multilabel immunofluorescence and confocal laser scanning microscopy (CLSM).

## 2. Material and Methods

### 2.1. Details of Patient Cohort

Post-mortem human brain tissue was obtained from the Netherlands Brain Bank (NBB, www.brainbank.nl, accessed on 1 March 2018) and the Department of Anatomy and Neurosciences of VU University Medical Center (VUmc, Amsterdam, The Netherlands). Written informed consent for brain autopsy and the use of the material and clinical information for research purposes had been obtained from the donor or the next of kin, in compliance with ethical and legal guidelines. Inclusion criteria for brain donors for the current study were (1) clinically diagnosed and pathologically confirmed PD or controls, without records of neurological or psychiatric disorders [3,20]; (2) availability of clinical records on clinical diagnosis, disease duration and presence and timing of parkinsonism and dementia; (3) availability of brain tissue samples with RIN > 6.0. The diagnosis of PD was based on the Movement Disorder Society diagnostic criteria [36]. Donors with known disease-causing mutations in genes associated with PD were excluded. Demographic features and clinical symptoms were extracted from the clinical files, including sex, age at symptom onset, age at death, disease duration, presence of dementia and time from disease onset until nursing home placement, as described previously [36].

Extensive neuropathological assessment was performed at the Netherlands Brain Bank. Braak and McKeith αSyn stages were determined using BrainNet Europe (BNE) criteria [37,38]. Braak neurofibrillary tangle (NFT) stages [37], CERAD neuritic plaque scores [39] and levels of AD-type pathology were determined according to NIA-AA consensus criteria [40] Additionally, the presence of aging-related tau astrogliopathy (ARTAG) [41] microvascular lesions and hippocampal sclerosis was assessed. APOE genotyping was performed using either the TaqMan^®^ SNP Genotyping Assay (Thermo Fisher Scientific, Waltham, MA, USA), or the Infinium^®^ NeuroChip Consortium Array (Illumina, San Diego, USA) (Supplementary Table S1).

### 2.2. mRNA Analysis of TLR 4

A total of 60 samples were analyzed by qPCR: per region (GTM and SN), we included 15 PD cases and 15 control donors (Supplementary Table S2). Total RNA was isolated with a Trizol^®^ Reagent (Invitrogen, Carlsbad, CA, USA)/chloroform protocol. RNA concentration and purity were determined using a NanoDrop ND-1000 spectrophotometer (Nanodrop Technologies, Wilmington, DE, USA) and RNA integrity was determined via the RNA integrity number (RIN) using an Agilent ^TM^ 2100 Bioanalyzer and an RNA 6000 Nano LabChip Kit (Agilent Technologies, Palo Alto, CA, USA).

The High Capacity cDNA Reverse Transcription Kit (catalogue n. 4368814, ThermoFisher, USA) was used for the cDNA synthesis, according to the manufacturer’s instructions. Per sample, 1.5 µg of RNA was used. The final volume after RT reaction was 150 µL.

TaqMan real-time PCR assays for TLR4 and SNCA genes and two reference genes (RPL30 and IPO8) were selected from the Thermo Fisher Scientific catalogue (Hs01060206_m1, Hs01103383_m1, Hs00265497_m1, Hs00183533_m1). The reference genes were selected for their consistent expression levels in previous experiments conducted with post-mortem brain material from both PD and control subjects. All qPCR reactions were prepared using TaqMan™Gene Expression master mix (catalogue n. 4369016, Thermo Fisher Scientific, USA) and were run with a Quant Studio 3 Real-Time PCR System (Thermo Fisher). Per reaction, 50 ng of cDNA was used, in a total volume of 20 µL. All samples were run in triplicate. The same thermal program, including a pre-cycling step (2′ at 50 °C + 10′ at 95 °C) and 40 amplification cycles (15″ at 95 °C + 1′ at 60 °C + 1′ at 65 °C), was used for all genes. The experiment was designed per gene, with all samples of the same region being placed into one plate; a negative control of the same region and a standard curve were included in each plate. The negative control was made by pooling together five samples, which underwent the retro-transcription reaction without the RT enzyme. A standard curve was generated for each assay run to calculate the efficiency of the qPCR reactions. The expression ratio of each gene of interest, relative to the geometric mean of the reference genes, was calculated by using the efficiency-corrected delta-delta Cq method [42]. The data of the qPCR were statistically analyzed with a Mann–Whitney U test and a *p*-value < 0.05 was considered significant.

### 2.3. Immunofluorescence and Confocal Microscopy

A total of 25 samples were analyzed by immunofluorescence: for the GTM region, we included 6 PD/PDD donors and 6 control donors; for the SN region, we included 6 PD/PDD donors and 7 clinically controlled cases, 3 of which were identified as iLBD at neuropathological examination (Supplementary Table S3).

Ten-micron-thick sections of paraffin-embedded tissue blocks were cut, deparaffinized and hydrated and processed for 30 min. The heat-induced antigen retrieval (HIER) step with citrate buffer pH 6.0 was performed in a steamer at >95 °C. For multilabeling immunofluorescence and confocal microscopy, thick paraffin-embedded sections were deparaffinized, rehydrated and incubated with 1% H_2_O_2_ for 30 min at room temperature (RT). After antigen retrieval with citrate buffer pH 6 for 30 min in a vegetable steamer, blocking was performed with 3% normal goat serum in 50 mM TBS; a cocktail of primary antibodies including polyclonal rabbit anti-TLR4 (LS-Bio, 141-42, dilution 1:200), polyclonal goat anti-Iba1 (Abcam, ab 5076, diluted 1:200) and mouse anti-phosphorylated Ser129-synuclein (11A5, courtesy of Prothena) diluted 1:20.000 [43] in blocking solution were incubated overnight at 4 °C in a humid chamber. Secondary fluorescence-conjugated antibodies (donkey-anti-mouse Alexa-fluor 488, Molecular Probes A21202 and donkey-anti-goat Alexa-fluor 594, Molecular Probes A11058) diluted 1:200 in blocking buffer were added to the sections that were incubated for 90 min at RT; after a new blocking step with 3% normal goat serum, the Envision HRP kit for rabbit antibodies (DAKO, K4003) was applied for 30 min at RT, followed by a 10 min incubation with Alexa 647 Tyramide (Alexa 647 Tyramide SuperBoost Kit, streptavidin, Invitrogen, B40936, dilution 1:100) to enhance the signal. For nuclear staining DAPI (Sigma, D9542, dilution 1:1000, 5 min incubation) was included and the other sections were mounted with Mowiol +DABCO. An overview of the demographic, clinical and pathological data of the IF donors selection is given in Table S1.

Confocal laser scanning microscopy was performed using a Leica TCS SP8 STED 3X microscope (Leica Microsystems). Images were collected using a 100 × 1.4 NA oil objective lens, with the resolution set to a pixel size of 20 nm × 20 nm. Gated hybrid detectors were used in counting mode. Sections were sequentially scanned for each fluorophore by irradiation with a pulsed white light laser at different wavelengths. After scanning, deconvolution was performed using CMLE (for confocal images) algorithms in Huygens Professional software (Scientific Volume Imaging, Huygens, The Netherlands). All images were adjusted for brightness/contrast in the same way using an ImageJ (National Institute of Health, Bethesda, MD, USA) script before image analysis. The presence of somatic pSer129 immunoreactivity was used to distinguish between cells with and without cytopathology.

## 3. Results

### 3.1. Quantification of TLR4 mRNA Levels

**Figure 1:**
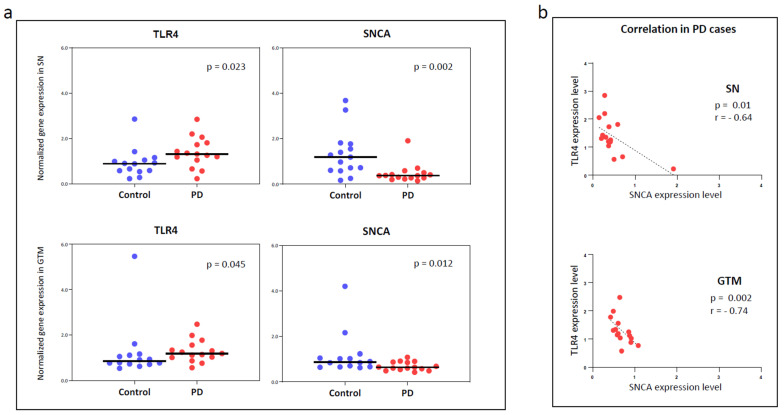
Gene expression quantification of TLR4 and alpha-synuclein in PD and control donors. (**a**) Real-time qPCR graphs show significant increase in TLR4 and decrease in alpha-synuclein normalized mRNA levels in SN and GTM brain regions of PD patients compared to control donors. (**b**) Negative correlation of TLR4 and alpha-synuclein gene expression levels in SN (*p* = 0.01, r = −0.64) and GTM (*p* = 0.002, r = −0.74) of PD patients.

To quantify the regional expression of TLR4 transcripts in postmortem brain tissue in an independent cohort of PD and control donors, we used relative quantitative RT-PCR. According to the data of this approach (Figure 1a), the expression levels of TLR4 were higher in the SN and GTM of PD than in age-matched controls. Specifically, a significant upregulation of TLR4 was found in the SN (+49.2%; *p* = 0.023) and GTM (+4.8%; *p* = 0.045) in PD in comparison with control donors. Meanwhile, the expression levels of αSyn were downregulated in both SN (−64.5%; *p* < 0.0028) and GTM (−41.7%; *p* < 0.0003) in PD. A negative correlation between TLR4 and α-Syn expression was observed in the SN (r = −0.64; *p* = 0.01) and GTM (r = −0.74; *p* = 0.002) in PD donors (Figure 1b). The correlations found in the entire cohort are shown in Figures S1 and S2.

### 3.2. TLR4 Co-Localizes with pSer129-αSyn in SN and GTM

**Figure 2:**
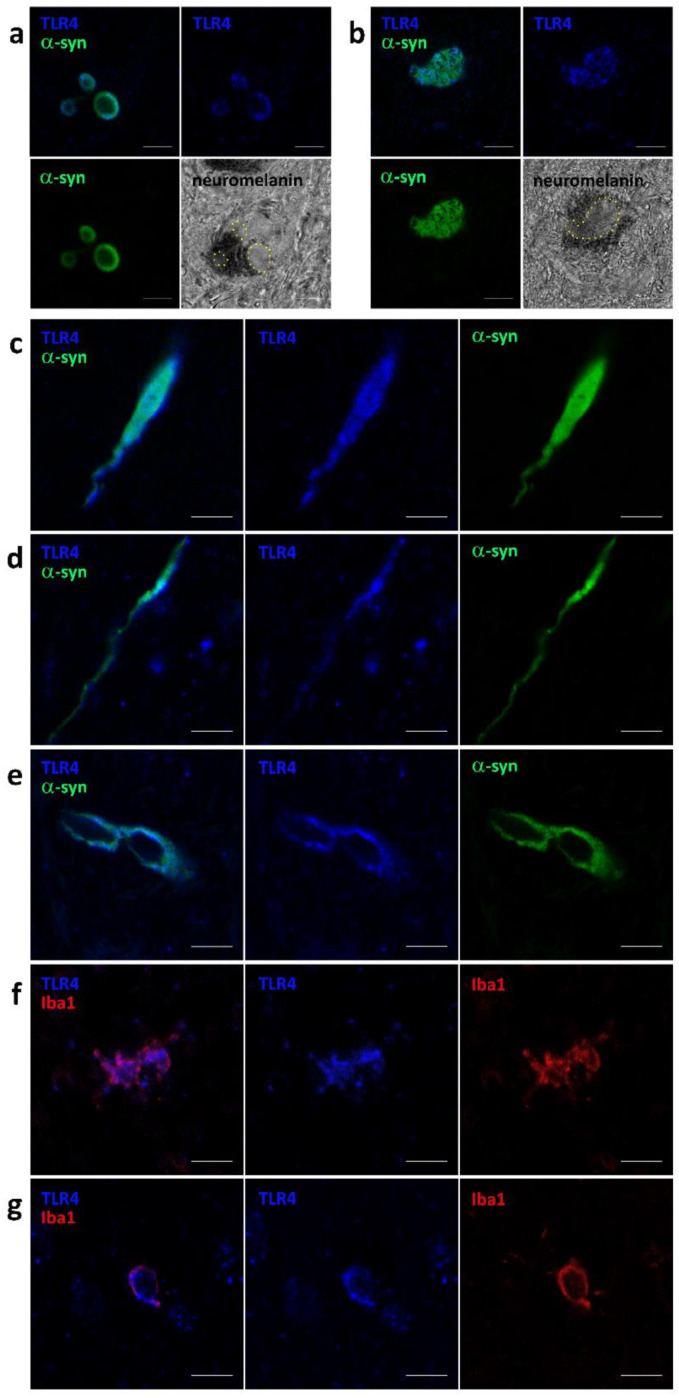
Confocal microscopy representative images of TLR4 co-localization with phospho-alpha-synuclein and Iba1 in substantia nigra and medial temporal gyrus of one iLBD (**a**,**c**,**d**) and two PD (**b**,**e**–**g**) donors. TLR4 (blue) and pSer129-αSyn (green) co-localize (**a**) in the periphery of Lewy bodies, (**b**) in pale bodies (outlined in yellow in the bright field images) of SN neuromelanin-containing neurons and in SN bulgy and thread-like neurites of iLBD (**c**,**d**) and PD (**e**) cases. TLR4 immunofluorescence is clearly detected also in cell body and processes of microglia (Iba1; red) in the SN (**f**) and GTM (**g**) of PD cases. Magnification 63 × 1.4. Scale bar: 20 μm.

To analyze the co-localization of TLR4 with pSer129-αSyn in Lewy bodies in PD (note controls did not have Lewy bodies), double labeling immunofluorescence was employed for TLR4 and pSer129-αSyn, again using tissue from the SN and GTM. We found co-localization of TLR4 with pSer129-αSyn in the periphery of Lewy bodies (Figure 2a, iLBD case) and in pale bodies (Figure 2b, PD case) of SN neuromelanin-containing neurons and, in the SN, we found bulgy and thread-like neurites indicative of iLBD (Figure 2c,d) and PD (Figure 2e) cases. We also confirm TLR4 immunoreactivity in microglia (iba1 positive) in the SN (Figure 2f) and GTM (Figure 2g) of PD cases.

The co-localization of TLR4 with αSyn and with Iba1 was also presented in 3D videos reported in the Supplementary Materials.

## 4. Discussion

Although it is not clear whether neuroinflammation precedes neurodegeneration in PD or if selective neuronal degeneration provokes a neuroinflammatory response, mounting evidence suggests that TLR-mediated immune responses can initiate inflammatory activity and contribute to neuronal loss that is responsible for PD onset and progression. The conversion to a pathological conformation is a feature of many proteins, including α-Syn, implicated in neurodegenerative disorders [44]. αSyn is linked genetically and neuropathologically to PD. In healthy cells, the proteostasis network serves to ensure the proper balance between synthesis, folding, trafficking and degradation of αSyn. Indeed, the physiological expression of αSyn plays a protective role against neuronal death. However, it is generally accepted that the dysfunction of cellular proteostasis is linked to PD and other neurodegenerative diseases [45]. Pathological aggregates of α-Syn result from the accumulation of misfolded proteins which cause ER stress [46], oxidative and nitrosative stress [47], and mitochondrial dysfunction [48].

TLR4 is of special interest in PD as αSyn has been shown to interact with TLR4, triggering an immune response that can cause αSyn aggregation, chronic inflammation and progressive neuronal injury [15,49,50]. Indeed, it has been suggested that aggregates of αSyn can act as DAMP-activating microglial cells through a mechanism that involves TLR upregulation [21].

Aggregates of α-Syn were reported to activate leucine-rich-repeat and NLR family pyrin domain-containing 3 of microglia through an interaction with TLRs, thus generating extensive microgliosis possibly caused by NF-κB-induced pro-inflammatory cytokine release [51,52].

In addition, several studies have demonstrated the alteration of TLR4 signaling in PD as well as increased levels of TLR4 in circulating monocytes and postmortem brain samples in PD patients compared to healthy controls [24,30,53]. Previously, using a 1-methyl-4-phenyl-1,2,3,6-tetrahydropyridine (MPTP) mouse model of PD, we demonstrated that the depletion of TLR4 was associated with the accumulation of αSyn and provided protection against MPTP toxicity [54]. In the same mouse model of PD, we showed that wild type mice were more vulnerable than TLR4-deficient animals to striatal dopamine depletion following MPTP intoxication [55]. Other authors, using in vivo models of PD, have highlighted a key role of TLR4 in mediating neuronal death, neuroinflammation and αSyn pathology [56,57,58]. On the contrary, TLR4 has proven effective in promoting αSyn clearance and protecting nigral dopaminergic neurons [20]. Therefore, the detrimental or beneficial effects of this innate immune receptor need to be investigated.

In this study, we aimed to examine the expression of TLR4 and link to αSyn pathology in post mortem brain tissues obtained from PD and control subjects. We found upregulation of TLR4 mRNA in both the SN and the GTM of PD cases compared to control subjects. This finding is consistent with those of other authors who have described the increased expression of TLR4 in the brain and blood of PD patients [24]. Increased TLR4 expression was also found in the putamen and frontal cortex [13,29] and was correlated with proinflammatory cytokine release, CD4^+^ infiltration and sustained microglia activation [31]. Here, we detected increased mRNA levels of TLR4 in the SN and GTM in post-mortem PD patients. Moreover, we found lower levels of αSyn in the SN and GTM of PD patients. This is in agreement with other studies showing that a decrease in αSyn levels in PD [59] is often associated with cognitive impairment [60,61,62,63]. A decrease in αSyn could be a result of neuronal and synaptic loss in PD patients or concomitant low levels of α-Syn-cleaving enzymes such as neurosin, calpain-1, cathepsin D and matrix metalloproteinase-3 [63,64]. Cleavage by proteases at specific sites of αSyn can affect the fate and function of the protein. This is an intriguing question that needs to be clarified.

Studies have reported that α-synuclein phosphorylated at Ser129 participates in PD pathogenesis [65,66,67,68]. About 90% of the total α-synuclein in LBs is phosphorylated at Ser129 [69,70]. However, a recent study shows that pS129–αSyn can inhibit αSyn fibril formation and seeded aggregation; therefore, the pathogenic relevance of pS129–αSyn remains controversial [71].

In this study, we aimed to assess the possible interplay between TLR4 and pSer129-αSyn-associated pathology by using immunofluorescence staining. We found immunoreactivity in LBs for TLR4 and pSer129-αSyn in neurons in the SN and GTM. An important finding is that the accumulation of pSer129-αSyn in cell bodies and in the processes of microglia in PD patients is associated with its co-localization with TLR4, suggesting a possible physical interaction between the two that could trigger an inflammatory response. This requires further future investigations.

Neuroinflammation is a crucial contributor to the pathogenesis of PD [72]. Several studies showed the presence of reactive microglia within the SN of PD patients and elevated amounts of pro-inflammatory molecules [73]. In addition, the existence of a functional link between αSyn and microglia has been reported. Indeed, αSyn engages TLRs on microglia leading to the activation of downstream signaling pathways, and nuclear translocation of NF-kB and the production of pro-inflammatory mediators [21,22].

Ionized calcium binding adaptor molecule 1 (Iba-1) is a marker of microglia activation. Studies have shown that the expression of Iba-1 is upregulated in the brain in a mouse model of PD [74]. In addition, LB-related αSyn aggregates were found in Iba1-immune reactive microglial cells of autopsy brain specimens from idiopathic PD patients [74]. Here, we detected Iba1-positive microglial cells in PD cases and co-localization between Iba1 and TLR4. This co-localization and the concomitant co-localization between TLR4 and pSer129-αSyn suggest also a proximity and interaction between TLR4 and αSyn that could be responsible for the microglial activation and inflammatory response in PD patients.

In conclusion, our findings support the possible involvement of TLR4 in PD pathogenesis and neuroinflammation. A deeper investigation of the molecular aspects of the interaction between TLR4 and pSer129-αSyn and between TLR4 and Iba-1 and the potential impact on αSyn aggregation could improve our understanding of the role of neuroinflammation in PD. The TLR4 pathway could be a mechanism-based therapeutic strategy for the treatment of PD.

## Data Availability

The data presented in this study are available on request from the corresponding authors.

## Supplementary Materials

The following supporting information can be downloaded at https://zenodo.org/record/7940727#.ZGRs4nKxWUm, Table S1: Demographics, clinical and pathological data of all cases. Table S2:Demographics, clinical and pathological data of qPCR cases. Table S3: Demographics, clinical and pathological data of immunofluorescence cases. Figure S1: Correlation for group of TLR4 and alpha-synuclein gene expression levels in SN. Figure S2: Correlation for group of TLR4 and alpha-synuclein gene expression levels in GTM. Supplementary videos: The videos correspond to the experiment shown in Figure 2. Video S1: large pale body in the SN of a PDD case displaying the co-localization between TLR4 (blue) and phospho-αSyn (green). The nuclei are represented as cyan circles while red color indicates the presence of glial cells; co-localization between TLR4 and Iba1 is shown in fuchsia. Video S2: co-localization of TLR4 (blue) and Iba1 (red) on the surface of a glial cell is shown in fuchsia. Nuclei are shown in cyan. Video S3: a LB with irregular in shape in the SN of an iLBD case, showing TLR4 (blue) overlapping with phospho-αSyn (green) next to a glial cells, in which Iba1 (red) overlaps with TLR4 (overlapping is shown in fuchsia). Video S4: a LB in the SN of an iLBD case, showing co-localization between TLR4 (blue) and phosph-αSyn. (nuclei are in cyan; glial cells are marked in red) Video S5: SN of a PDD case: at the bottom-right, an LB with a clear co-localization between TLR4 (blue dots)_ and aSyn (green); in the center-right, and upper-left, two glial cells showing colocalization between Iba1 (red) and TLR4 (blue). Nuclei are shown in cyan. Video S6: large pale body in the SN of a PDD case with clear blue-green staining indicating TLR4 and phospho-αSyn co-localization. Video S7: large LB in the GTM of a PD case with clear blue-green staining indicating TLR4- phospho-αSyn co-localization.
